# Extensive Placental Abruption With Fetal Demise and Couvelaire Uterus in an Adolescent in a Low-Resource Setting: A Case Report

**DOI:** 10.7759/cureus.119569

**Published:** 2026-11-12

**Authors:** Myung S Choi, Charlotte R Laidler, Katherine H Gonzalez Valladares, Sorivel Sosa, Katherin A Gonzalez

**Affiliations:** 1 Biology, Syracuse University, Syracuse, USA; 2 Biology, Columbia University, New York City, USA; 3 Chemistry, Texas Tech University, Lubbock, USA; 4 Research, Global Health Leaders, Bend, USA; 5 Obstetrics and Gynecology, Clínica Dres. Arache Poueriet, Hato Mayor del Rey, DOM

**Keywords:** adolescent pregnancy, fetal demise, massive obstetric hemorrhage, maternal near-miss, placental abruption

## Abstract

Placental abruption is a life-threatening obstetric emergency with disproportionate impact on adolescent populations, who carry elevated biological risk for hypertensive complications and poor perinatal outcomes. This case report describes a 17-year-old primigravida from the Dominican Republic who presented at 34 weeks of gestation with severe abdominal pain, decreased fetal movements, and absent fetal heart tones on auscultation. Admission ultrasonography was reported to show fetal demise and placental findings described as infarction. Emergency cesarean delivery revealed approximately 90% placental separation and hemorrhagic infiltration described as involving approximately 20% of the posterior uterine surface, consistent with Couvelaire uterus. Estimated blood loss was approximately 1300 mL, although the period covered by this estimate was not documented in the available records. Postoperative investigations showed elevated lactate dehydrogenase (1027 U/L), thrombocytopenia (86 × 10⁹/L), severe anemia (hemoglobin 5.6 g/dL), mildly elevated aspartate aminotransferase (61 U/L), and renal dysfunction (creatinine 2.99 mg/dL). These findings raised concern for possible hemolysis, elevated liver enzymes, low platelets (HELLP) syndrome, but the available evidence was insufficient to confirm complete or partial HELLP syndrome. Supporting postoperative blood pressure measurements were not documented in the available records, preventing independent confirmation of preeclampsia. The patient reported five antenatal visits, but their timing and clinical content were not documented in the available records; therefore, the adequacy of antenatal surveillance could not be established. The specific indication for cesarean delivery after fetal demise, transfusion quantities, and subsequent laboratory trends and maternal outcomes were also not documented in the available records. A qualifying WHO maternal near-miss criterion could not be verified. This case describes extensive placental abruption with fetal demise and Couvelaire uterus and highlights the importance of explicit diagnostic criteria, consistent clinical documentation, and continuity of information across care settings. The available evidence does not establish the cause or preventability of this patient's complications.

## Introduction

Placental abruption, defined as the premature separation of the placenta from its uterine attachment before delivery, is a life-threatening obstetric emergency that complicates approximately 0.6% to 1.2% of all pregnancies [1]. The clinical manifestations typically include sudden-onset vaginal bleeding, abdominal pain, and abnormal fetal heart rate patterns. The underlying pathophysiology of abruption is driven by a complex interaction between acute mechanical triggers and long-standing chronic processes, such as uteroplacental vasculopathy, systemic inflammation, and ischemic placental disease [1]. These chronic conditions often lead to defective spiral artery remodeling and placental hypoperfusion weeks or months before the abruption becomes clinically evident. When a severe detachment occurs, it forces the release of excess thrombin and pro-inflammatory cytokines into the maternal bloodstream, rapidly precipitating profound maternal hemorrhage, consumptive coagulopathy, and fetal demise [1].

Because placental abruption is often the culmination of chronic placental dysfunction, early and consistent prenatal care is vital for identifying at-risk individuals and mitigating potential complications [1]. Unfortunately, comprehensive prenatal monitoring is frequently inaccessible in resource-limited settings like the Dominican Republic, where hypertensive disorders like preeclampsia and eclampsia remain the leading causes of maternal mortality [2]. Inadequate prenatal care remains a major barrier to preventing maternal morbidity; pregnant individuals often delay seeking medical attention until the second half of gestation, missing the critical early-pregnancy window required to assess risk [2]. This lack of intervention is especially detrimental to adolescent populations, who carry a significantly increased risk for poor obstetric outcomes due to biological immaturity and socioeconomic vulnerability [3]. The current case is particularly relevant given the high prevalence of adolescent pregnancy in the Dominican Republic. The country's adolescent pregnancy rate remains far above the regional average, with approximately 22% of Dominican adolescents having experienced pregnancy [4,5]. Adolescent pregnancies are frequently associated with higher risks of hypertensive disorders and premature delivery, yet there is a notable research gap regarding severe maternal morbidity and "near-miss" outcomes in the country [6]. Furthermore, stillbirth remains a globally neglected research area, with a significant lack of data from low- and middle-income countries where the burden is highest [7].

This report presents the case of a 17-year-old patient from the Dominican Republic with extensive placental abruption, fetal demise, and Couvelaire uterus. Postoperative laboratory abnormalities raised concern for hemolysis, elevated liver enzymes, low platelets (HELLP) syndrome, but preeclampsia and complete or partial HELLP syndrome could not be confirmed from the available records. The report describes the clinical findings and the limitations of diagnostic interpretation when documentation is incomplete.

## Case presentation

Patient information

The patient's name was withheld to protect her privacy. She was a 17-year-old Dominican female of Mestizo ethnicity with a B-positive blood type. She was a student and lived with her partner in a common-law marriage.

The patient presented to the hospital at 34 weeks of gestation with severe abdominal pain. Fetal heart tones were not audible on auscultation, and she reported decreased fetal movements. The duration of decreased fetal movements was not documented in the available records. Obstetric ultrasonography was performed during the admission assessment and was reported to show a gestational age of 34 weeks and placental findings described as infarction. Although placental abruption, labor, and uterine rupture are relevant differential considerations for this presentation, the treating team's contemporaneous differential diagnosis was not documented in the available records. Detailed sonographic findings were also not documented in the available records. Placental abruption was subsequently confirmed intraoperatively. Family and psychosocial histories and information concerning genetic risk factors were not documented in the available records.

The patient was a primigravida with no reported history of surgery. She reported attending five antenatal visits at another hospital. The gestational ages at these visits, blood pressure measurements, laboratory results, and other clinical assessments were not documented in the available records. Consequently, the timing and adequacy of antenatal care could not be established. The patient provided an ultrasound image from a previous appointment. Its date, gestational age at examination, and accompanying ultrasound report were not documented in the available records; therefore, the image could not establish the fetus's previous clinical condition or the timing of placental pathology.

This sonography was provided by the patient from a previous appointment of an unknown date. It shows the patient's fetus is healthy with no physical deformities or visualizations indicative of a hemorrhage. The head of the fetus is visible on the left side of the image, and the spine is seen in the upper middle portion of the image (Figure 1). This sonography indicates that the fetus was healthy without signs of a potential hemorrhage at the time of assessment, yet it is difficult to decipher its proximity to the patient's current hospital visit without a date for the sonography.

**Figure 1:**
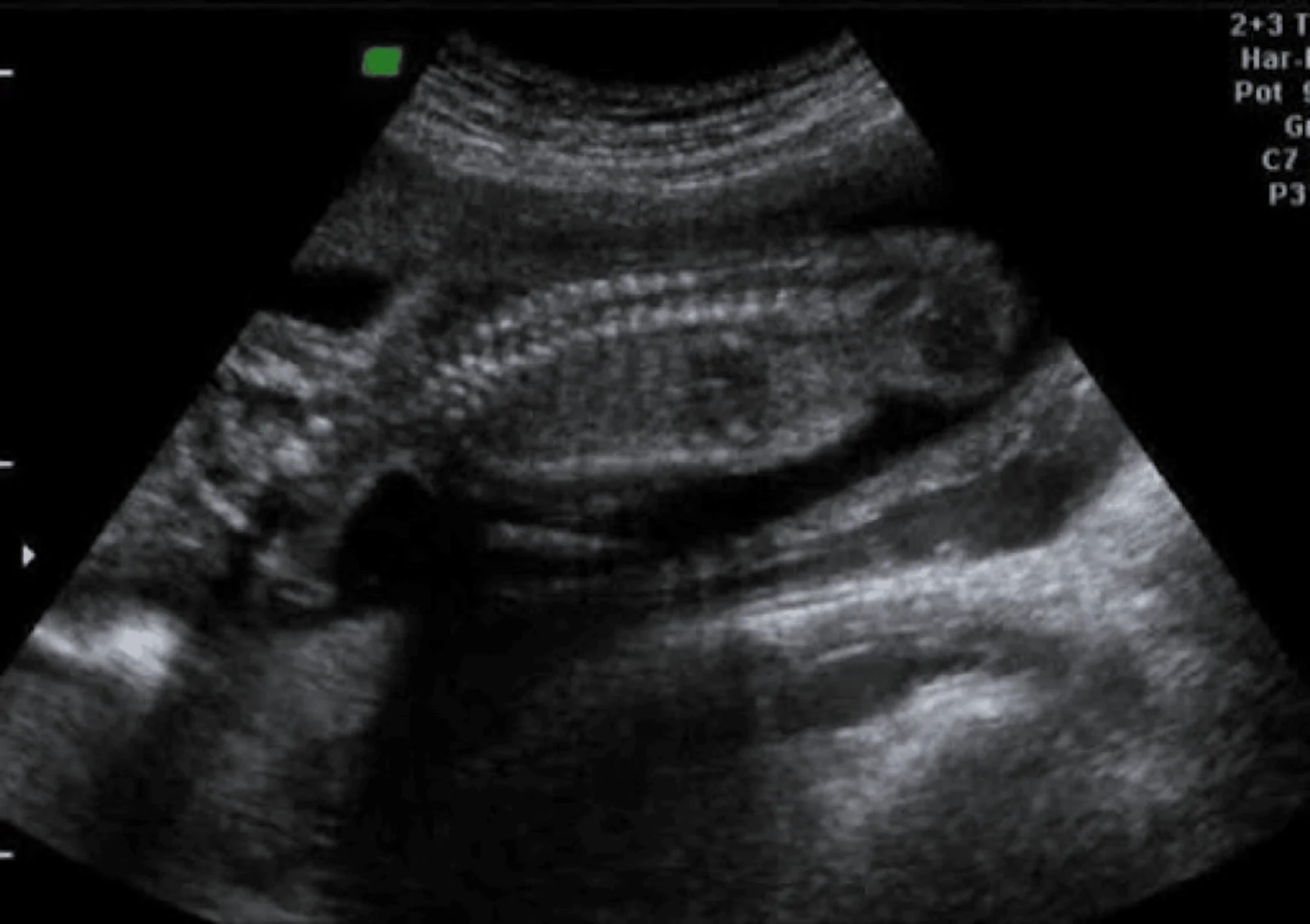
Sonography of the patient's womb in a previous appointment

Her first menstrual cycle was at 12 years old, with breast development beginning at 11 years old alongside the onset of puberty. Her menstrual history was noted to have a normal 21-35 day cycle, with a four-day bleeding period, where she bled ~40 mL per day. The patient denied providing any additional surgical or medical history, but noted that she consumes coffee and alcohol on occasion.

Clinical findings

The patient complained of severe abdominal pain and decreased fetal movements. Fetal heart tones were not audible on auscultation. During examination in the emergency room, she was conscious and oriented and continued to complain of pain. Recorded vital signs were blood pressure 110/70 mmHg, heart rate 110 beats/min, respiratory rate 20 breaths/min, temperature 37.5°C, and oxygen saturation 98%.

On physical examination, the head was normocephalic, with no palpable masses or bony depressions. The neck was mobile, with a palpable carotid pulse and no palpable lymphadenopathy. The extremities were symmetrical and mobile, with peripheral pulses present. The chest had symmetrical expansion. Tanner staging was not documented in the available records. Vesicular breath sounds were present without crackles, and heart sounds were normal, with no audible murmurs. The abdomen was enlarged by the gravid uterus, with a single fetus in a longitudinal lie and cephalic presentation. Fundal height measured 29 cm, and no fetal movements were observed.

Uterine activity was inconsistently documented: one entry recorded four contractions per 10 minutes, whereas another recorded no contractions over 10 minutes. The timing of these observations relative to each other was not documented in the available records, so it could not be determined whether this reflected a clinical change or a documentation discrepancy.

During emergency cesarean delivery, a single fetus was delivered without signs of life. Approximately 90% placental separation was observed, together with hemorrhagic infiltration described as involving approximately 20% of the posterior uterine surface, consistent with Couvelaire uterus. The uterus was soft and had poor contractility. Estimated blood loss was approximately 1300 mL. Whether this estimate included blood loss before surgery or during the postoperative period was not documented in the available records. The uterus was temporarily exteriorized, massaged, and returned to the abdominal cavity. Resolution of the hemorrhagic infiltration was not documented in the available records.

The specific maternal indication for cesarean delivery after fetal demise, the decision-to-delivery interval, and whether induction or a trial of labor was considered or attempted were not documented in the available records. Postoperative blood pressure and heart rate measurements and their timing were also not documented in the available records; therefore, postoperative hemodynamic trends could not be assessed.

Postoperative investigations included complete blood count, blood glucose, uric acid, lactate dehydrogenase (LDH), serum albumin, total proteins, blood urea nitrogen, creatinine, aspartate aminotransferase (AST), alanine aminotransferase (ALT), total bilirubin, direct bilirubin, indirect bilirubin, prothrombin time, activated partial thromboplastin time, and urinalysis. The available documentation described pending blood cross-matching and a plan for packed red blood cell transfusion. The number and type of blood products actually administered, their timing, and the post-transfusion laboratory response were not documented in the available records.

Table 1 shows several clinically important abnormalities, including severe anemia, thrombocytopenia, elevated LDH, and renal dysfunction. LDH was 1027 U/L, compared with the reference range of 140-280 U/L listed in the table. The platelet count was 86 × 10⁹/L, hemoglobin was 5.6 g/dL, hematocrit was 15.9%, and creatinine was 2.99 mg/dL. AST was 61 U/L, and ALT was 40 U/L. Total, direct, and indirect bilirubin were within the listed reference ranges. Although the laboratory abnormalities raised concern for HELLP syndrome, they did not independently confirm the diagnosis. Severe anemia occurred in the setting of documented hemorrhage, and elevated LDH alone did not establish hemolysis. Serial laboratory results and the post-transfusion response were not documented in the available records.

**Table 1:** Results of the patient's labs taken post-surgery

Laboratory parameter	Patient Value	Unit	Reference range
Hemoglobin (Hgb)	5.6	g/dL	12.0–16.0
Hematocrit (Hct)	15.9	%	36–46
Platelet count (Plt)	86	×10³/mm³	150–450
White blood cell count (WBC)	16.1	×10³/mm³	4.0–10.0
Prothrombin time (PT)	16	s	11–13.5
Activated partial thromboplastin time (aPTT)	37	s	25–35
Albumin	2.8	g/dL	3.5–5.0
Direct bilirubin (DB)	0.1	mg/dL	0.0–0.3
Indirect bilirubin (IB)	0.3	mg/dL	0.2–0.8
Total bilirubin (TBIL)	0.4	mg/dL	0.1–1.2
Creatinine (Cr)	2.99	mg/dL	0.6–1.2
Aspartate aminotransferase (AST)	61	U/L	10–40
Alanine aminotransferase (ALT)	40	U/L	7–56
Lactate dehydrogenase (LDH)	1027	U/L	140–280
Total protein (TPT)	4.2	g/dL	6.0–8.3
Blood urea nitrogen (BUN)	27	mg/dL	7–20
Uric acid	6.2	mg/dL	2.4–6.0
Sodium (Na)	141	mEq/L	135–145
Potassium (K)	4.5	mEq/L	3.5–5.0
Chloride (Cl)	110	mEq/L	98–106

Timeline

Diagnostic Assessment

The diagnostic assessment was based on the patient's clinical presentation, reported imaging findings, intraoperative observations, and laboratory results (Figure 2). At admission at 34 weeks of gestation, she reported severe abdominal pain and decreased fetal movements, with absent fetal heart tones on auscultation. Admission ultrasonography was reported to show fetal demise and placental findings described as infarction. Specific sonographic signs of abruption were not documented in the available records. Extensive placental abruption was confirmed intraoperatively, with approximately 90% placental separation and findings consistent with Couvelaire uterus. Hemorrhagic infiltration was described as affecting approximately 20% of the posterior uterine surface. The method of estimation and any standardized grading criteria were not documented in the available records.

**Figure 2:**
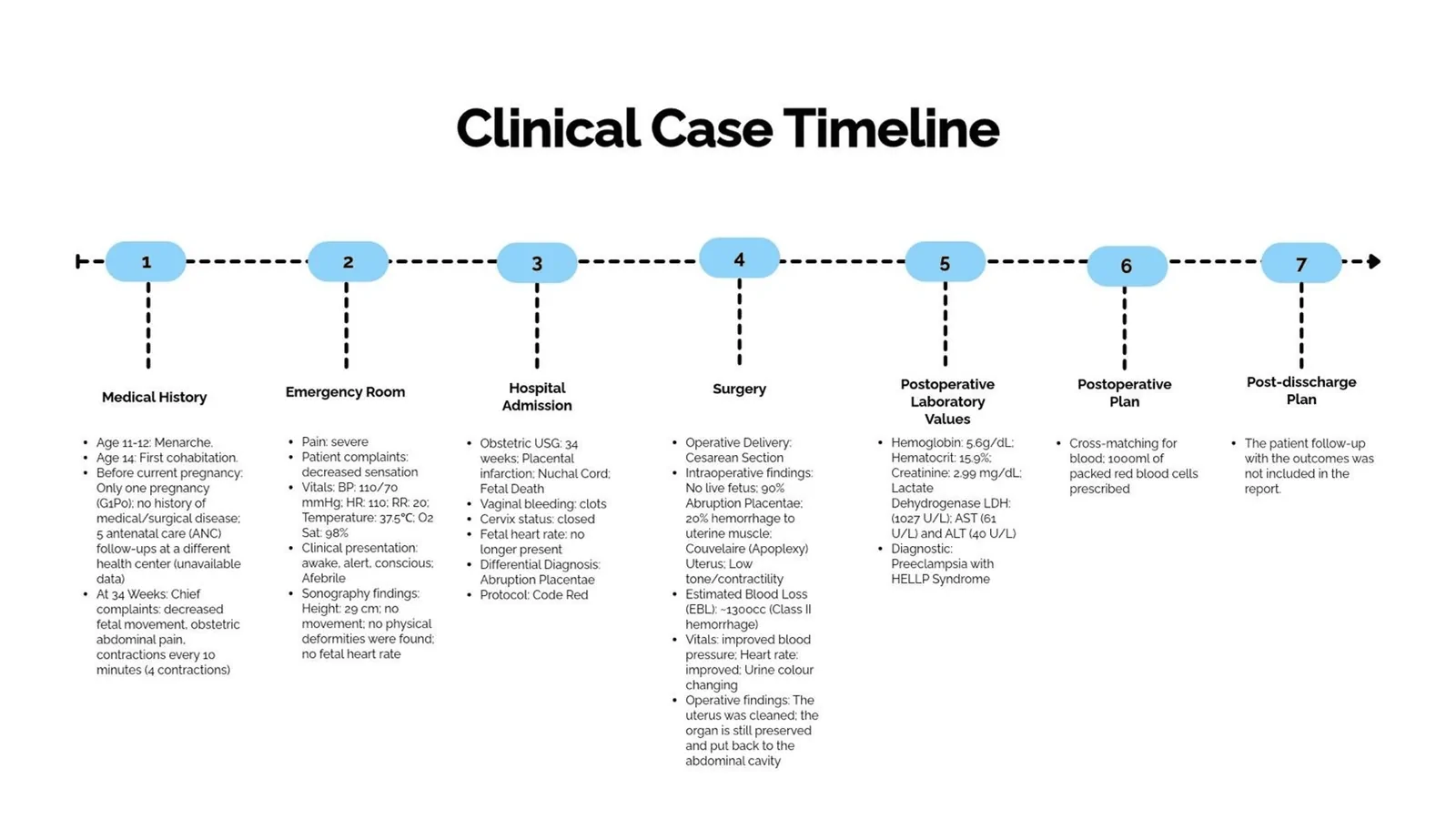
Clinical case timeline LDH - lactate dehydrogenase; AST - aspartate aminotransferase; ALT - alanine aminotransferase; HELLP - hemolysis, elevated liver enzymes, low platelet

Postoperative laboratory findings raised concern for possible partial HELLP syndrome, including elevated LDH of 1027 U/L and thrombocytopenia of 86 × 10⁹/L. However, elevated LDH alone did not establish hemolysis. AST was 61 U/L, ALT was 40 U/L, and bilirubin values were within the listed reference ranges. The available findings were insufficient to confirm HELLP syndrome or the proposed partial presentation in the context of diagnostic guidance on hypertensive disorders of pregnancy [8]. Additional evidence establishing hemolysis and serial laboratory trends was not documented in the available records. The admission blood pressure was 110/70 mmHg, and supporting postoperative blood pressure measurements were not documented in the available records; therefore, the proposed diagnosis of preeclampsia could not be independently verified.

Additional findings included severe anemia, with hemoglobin 5.6 g/dL, and renal dysfunction, with creatinine 2.99 mg/dL. The available documentation was insufficient to confirm a qualifying WHO maternal near-miss criterion. Relevant differential considerations included labor and uterine rupture for the presenting symptoms, and blood-loss anemia and hemorrhage-associated renal dysfunction for the postoperative abnormalities. The treating team's contemporaneous differential diagnosis and complete assessment of alternative causes were not documented in the available records. Alternative diagnoses therefore could not be definitively excluded from the available documentation. Although the patient reported five antenatal visits, their timing and clinical content were not documented in the available records. An antecedent untreated hypertensive disorder, the adequacy of antenatal care, and the potential benefit of a specific preventive intervention could not be established.

Therapeutic Intervention

The patient underwent emergency cesarean delivery. The specific maternal indication and rationale for choosing cesarean rather than vaginal delivery were not documented in the available records. The operation began with a 10-12 cm transverse suprapubic Pfannenstiel incision, followed by dissection of the subcutaneous tissue and transverse opening of the aponeurosis. The subaponeurotic muscular plane was then dissected, beginning at the midline, with attention to hemostasis of perforating vessels. The rectus abdominis muscles were separated vertically until the parietal peritoneum was identified. The peritoneal cavity was entered, and the lower uterine segment was identified. A Kerr-type uterine incision was made, and a single fetus was delivered without signs of life.

Approximately 90% placental separation was observed, together with hemorrhagic infiltration described as involving approximately 20% of the posterior uterine surface, consistent with Couvelaire uterus. The uterus was soft and had poor contractility. Estimated blood loss was approximately 1300 mL. Whether this estimate included blood loss before surgery or during the postoperative period was not documented in the available records.

The uterus was temporarily exteriorized, massaged, and returned to the abdominal cavity. Objective changes in uterine tone following massage and resolution of the hemorrhagic infiltration were not documented in the available records. The incision was closed in anatomical layers, and the patient was transferred to the recovery room.

## Discussion

The pathophysiology of the clinical cascade

Placental abruption is frequently the acute manifestation of long-standing chronic placental dysfunction, driven by defective spiral artery remodeling, systemic inflammation, and ischemic placental disease [1,9]. This underlying poor placentation may involve a failure of maternal immune tolerance to the fetal allograft, an immunological phenomenon often observed in primigravida pregnancies [9,10]. The patient reported five antenatal visits, but their timing and clinical content were not documented in the available records; therefore, the adequacy of antenatal surveillance could not be established. The sFlt-1/PlGF ratio has been investigated in women with suspected preeclampsia, rather than as an established predictor of placental abruption [11]. Furthermore, point-of-care approaches are being developed to improve access to biomarker testing for preeclampsia [12]. Low-dose aspirin initiated between 11 and 14 weeks of gestation has reduced preterm preeclampsia in screened high-risk pregnancies [13]. However, this patient's eligibility for prophylaxis, its use, and its potential effect on her outcome could not be established from the available records.

The surgical findings (approximately 90% placental separation, Couvelaire uterus with hemorrhagic infiltration described as affecting approximately 20% of the posterior uterine surface, and estimated blood loss of 1,300 mL) demonstrate the severity of the presentation but do not establish preceding chronic placental dysfunction in this patient.

Postoperative findings and possible HELLP syndrome 

Preeclampsia and its severe complications can develop or be recognized for the first time during the early postpartum period, making postoperative monitoring important [8]. However, supporting postoperative blood pressure measurements were not documented in the available records, and preeclampsia could not be independently confirmed in this patient.

The pathophysiology of preeclampsia and HELLP syndrome involves maternal endothelial dysfunction and systemic inflammation [8], with hepatic involvement potentially causing hepatocellular injury [14]. Severe early-onset HELLP presentations have also been described, although the review cited here concerns pregnancies before 23 weeks and is not directly comparable to this presentation at 34 weeks [15]. This patient had elevated LDH, mildly elevated AST, severe anemia, and thrombocytopenia, whereas ALT and bilirubin values were within the listed reference ranges (Table 1). These findings raised concern for possible HELLP syndrome but did not establish complete or partial HELLP syndrome or prove a specific mechanism of postoperative deterioration.

Adolescent vulnerability and severe maternal morbidity

This case involved severe obstetric complications, but a qualifying WHO maternal near-miss criterion could not be verified from the available records. Institutional quality of care is an important consideration in maternal outcomes in the Dominican Republic [6]. Preeclampsia and eclampsia in adolescent pregnancy are also important concerns, with variation across populations and settings [3].

Documenting this 17-year-old patient's complications provides an opportunity to discuss broader healthcare and socioeconomic challenges [6]. In the Dominican Republic, adolescent pregnancy occurs within a complex context of educational, social, and economic factors [5]. However, their contribution to this individual patient's outcome could not be established. The case should therefore be interpreted alongside broader evidence concerning institutional care and maternal outcomes, rather than as proof of systemic failures in this patient's care [6].

Recommendations for lower-resource areas

Data Openness and Collaborative Research

To effectively address the profound obstetric risks of adolescent pregnancy, there must be a concerted effort toward data openness and information sharing within the global academic community. Currently, there is a severe lack of data regarding stillbirths and severe maternal morbidity in low- and middle-income countries, contributing to a "10/90 gap" where less than 10% of research resources target conditions affecting over 90% of the global population [7]. The DR serves as a poignant example of this challenge [2]. While the Ministry of Public Health has developed high-quality treatment protocols using the GRADE methodology, these guidelines are often based on international evidence rather than robust national data that reflects local clinical realities [2]. This disconnect is further widened by a failure to effectively disseminate available information to frontline staff, chronic shortages of essential medicines and equipment, and an institutional culture where senior staff often resist new evidence-based standards in favor of traditional habits [2]. Also, even with proper dissemination of information, providers may not be fully informed of a patient's family and psychological history and/or any previous appointments regarding their health. Family and psychosocial histories and the details of the five reported antenatal visits were not documented in the available records. Whether further information was accessible through another clinical information system was also not documented in the available records. This leaves providers without crucial information needed to make more timely and informed decisions for their patients. Ultimately, improving care for this vulnerable population requires establishing open registries for maternal outcomes to identify these systemic barriers and finally align national policy with the reality of patient care [2].

Implementation of Clinical Practice Guidelines (CPGs)

Evidence-based clinical guidelines must be universally adopted. In the DR, the acceptability of CPGs is recognized by health professionals, but implementation is consistently hindered by a severe lack of necessary medicines, supplies, and equipment [2]. Removing barriers to the supply and use of essential, life-saving medications, such as magnesium sulfate for eclampsia prophylaxis, must be a priority for local and international health agencies [16].

Task-Shifting and Community-Based Care

Where specialist care is scarce, task-shifting allows community or lower-level health workers to administer critical prophylactic interventions [8]. To ensure patients actually reach the hospital, the health system must address the information gap through aggressive community-based messaging and education [12]. Many patients in low-resource settings have a limited comprehension of the disease's nature, often failing to seek care until after 20 weeks or when symptoms have already become severe [12]. Systemic barriers, including prohibitive transport costs and the vast distances to referral centers, frequently prevent women from following through on medical recommendations [12]. Bridging this gap requires not only the mobilization of community health workers to monitor "danger signs" but also the adaptation of clinical guidelines into simple, accessible models that patients and their families can actually understand [2].

Early Warning Systems and Quality Monitoring

Hospitals should implement a Modified Obstetric Early Warning System (MOEWS) to better identify and monitor deteriorating patients in understaffed and overcrowded environments [6]. Systematically registering maternal near-misses and severe maternal morbidity will help institutions gather patient feedback and identify precise gaps in emergency obstetric care [6].

Public Health and Socioeconomic Interventions

Structural interventions must address the root socioeconomic causes of adolescent vulnerability. Expanding access to modern contraceptives and implementing comprehensive sexual and reproductive health education are essential [5]. Furthermore, broader social protection programs have proven effective; in the DR, municipal participation in the Jornada Escolar Extendida (Extended School Day) ensures girls remain supervised and in school, while the Comer es Primero program incentivizes antenatal care, both of which are statistically associated with a lower probability of late adolescent pregnancy [5].

## Conclusions

This case describes extensive placental abruption, fetal demise, and a Couvelaire uterus in an adolescent patient. She reported five antenatal visits, but their timing and clinical content were not documented in the available records; therefore, the adequacy of antenatal surveillance could not be established. Although timely risk identification and antenatal follow-up are important, the available evidence does not establish whether earlier intervention would have altered this patient's outcome.

Preventing similar cases will require more than clinical recognition alone. Standardized near-miss registries, such as those leveraging the Latin American Center for Perinatology, Women and Reproductive Health (Red CLAP) network and the Perinatal Information System (SIP)-Plus database, could help generate the national-level evidence needed to guide policy and adapt protocols to local realities. However, maximizing the utility of these existing regional platforms requires addressing barriers such as inconsistent reporting, systemic resource limitations, and data fragmentation that currently constrain their full integration into routine clinical practice. At the bedside, existing guidelines must be paired with the resources, staffing, and task-shifting frameworks needed for consistent implementation, including community-level access to prophylactic care, though pharmacologic prophylaxis such as low-dose aspirin requires individual risk assessment and appropriate clinical oversight. Progress will depend on open data systems that make maternal near-miss outcomes visible, alongside sustained investment in the education, contraceptive access, and socioeconomic conditions that determine whether adolescents reach antenatal care in the first place.
